# Aptamers targeting amyloidogenic proteins and their emerging role in neurodegenerative diseases

**DOI:** 10.1016/j.jbc.2021.101478

**Published:** 2021-12-09

**Authors:** Kazuma Murakami, Naotaka Izuo, Gal Bitan

**Affiliations:** 1 Division of Food Science and Biotechnology, Graduate School of Agriculture, Kyoto University, Kyoto, Japan; 2 Laboratory of Pharmaceutical Therapy and Neuropharmacology, Faculty of Pharmaceutical Sciences, University of Toyama, Toyama, Japan; 3 Department of Neurology, David Geffen School of Medicine, Brain Research Institute, and Molecular Biology Institute, University of California Los Angeles, Los Angeles, California, USA

**Keywords:** oligonucleotide, RNA, DNA, amyloid, Alzheimer's disease, Parkinson's disease, prion, blood-brain barrier, oligomer, exosome, αSyn, α-synuclein, Aβ, amyloid β-protein, AD, Alzheimer's disease, ADDLs, Aβ-derived diffusible ligands, AI, artificial intelligence, CD, circular dichroism, HT-SELEX, high throughput-systematic evolution of ligands by exponential enrichment, PD, Parkinson's disease, PICUP, photo-induced cross-linking of unmodified protein, RVG, rabies viral glycoprotein, SDS-PAGE, sodium dodecyl sulfate–polyacrylamide gel electrophoresis, SELEX, systematic evolution of ligands by exponential enrichment

## Abstract

Aptamers are oligonucleotides selected from large pools of random sequences based on their affinity for bioactive molecules and are used in similar ways to antibodies. Aptamers provide several advantages over antibodies, including their small size, facile, large-scale chemical synthesis, high stability, and low immunogenicity. Amyloidogenic proteins, whose aggregation is relevant to neurodegenerative diseases, such as Alzheimer’s, Parkinson’s, and prion diseases, are among the most challenging targets for aptamer development due to their conformational instability and heterogeneity, the same characteristics that make drug development against amyloidogenic proteins difficult. Recently, chemical tethering of aptagens (equivalent to antigens) and advances in high-throughput sequencing-based analysis have been used to overcome some of these challenges. In addition, internalization technologies using fusion to cellular receptors and extracellular vesicles have facilitated central nervous system (CNS) aptamer delivery. In view of the development of these techniques and resources, here we review antiamyloid aptamers, highlighting preclinical application to CNS therapy.

Neurodegenerative diseases are characterized by a progressive loss of neuronal function. Most of these diseases are age-related, and self-assembly of amyloidogenic proteins is thought to be a cause or a major deleterious mechanism in many of them. Examples include Alzheimer’s disease (AD) and various other tauopathies, Parkinson’s diseases (PD) and other synucleinopathies, prion diseases, and many other sporadic or genetic proteinopathies. The amyloidogenic proteins involved in these diseases are prone to self-association into neurotoxic oligomers and amyloid fibrils (Fig. 1). In many cases, the oligomers, which have metastable structures, have been shown to play pivotal roles in the pathogenesis of the associated diseases and to be more toxic than the structurally stable fibrils (1). The abnormal protein assemblies cause neurotoxicity by a variety of mechanisms, including apoptosis, oxidative stress, inflammation, and disruption of proteostasis through blockage of proteasomal and lysosomal protein degradation. Given the significance of oligomers in the pathogenesis of many neurodegenerative diseases (2, 3, 4), they have been the focus of attention as molecular targets of both diagnostic and therapeutic research and development. Therefore, generation of specific oligomer-binding reagents is a promising approach for development of early detection tools and redirecting the self-assembly process into dissociation back to nontoxic monomers, formation of nontoxic and nonamyloidogenic assemblies amenable to degradation, or in some cases, accelerating the aggregation process to reduce steady-state levels of oligomers in favor of less-toxic fibrils.

**Figure 1:**
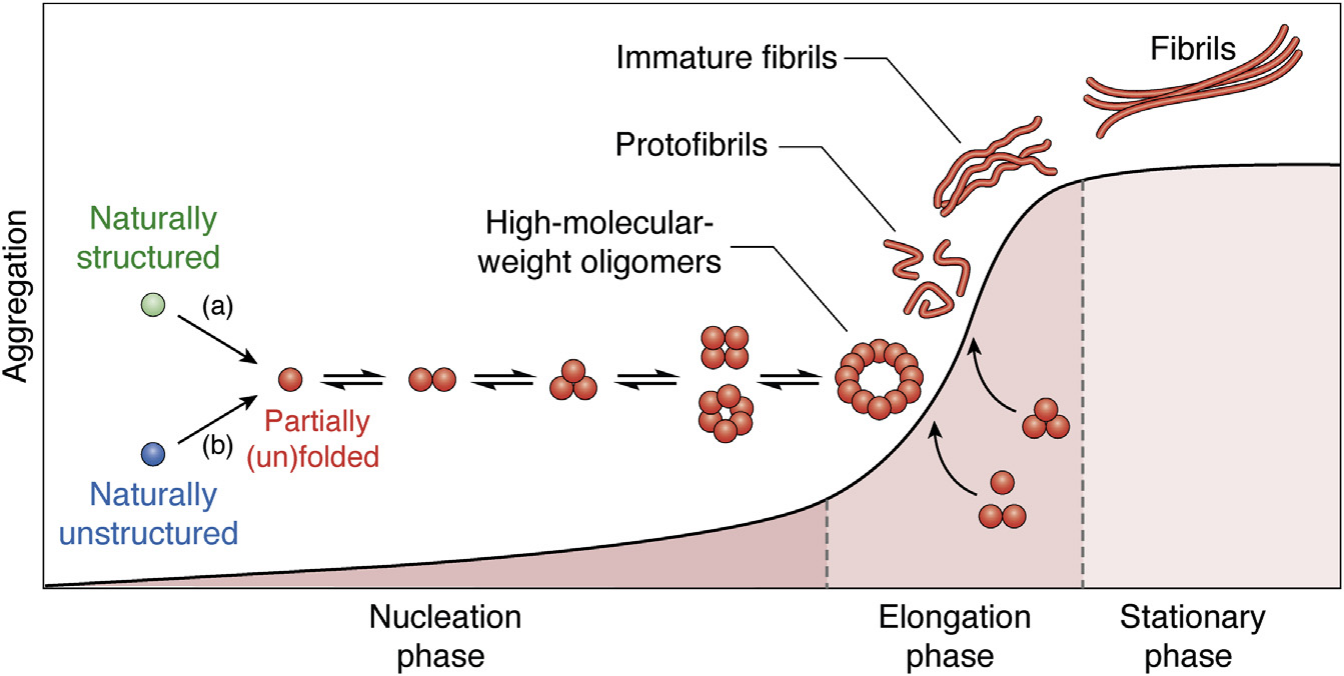
**A Schematic diagram of amyloidogenic proteins aggregation.** Amyloidogenic proteins can be naturally structured or unstructured. Naturally structured proteins (a), *e.g.*, PrP undergo partial unfolding, whereas naturally unstructured proteins (b), such as Aβ, αSyn, or tau, undergo partial folding under pathological conditions, initiating the self-assembly process. Both cases lead to formation of partially (un)folded monomers, which self-associate into increasing-size oligomers until a quasi-stable nucleus forms leading to the elongation phase. Elongation typically proceeds at a fast rate compared with the nucleation and may involve formation of quasi-stable high-molecular-weight oligomers, protofibrils, and eventually fibrils. Finally, the monomers are consumed and the system reaches a stationary phase in which no more growth is observed.

Aptamers are molecular-recognition agents comprising single-stranded DNA or RNA oligonucleotides that similar to antibodies, bind specifically to diverse targets, including small molecules, peptides, proteins, and nucleic acids (5). Amyloidogenic proteins inherently tend to bind nucleic acids (6, 7, 8) making it difficult to select aptamers specific for just one protein and even more so for distinct assembly states of these proteins. Nonetheless, aptamers offer several advantages compared with antibodies including their small size, facile chemical synthesis, including in large scale, high stability, and low immunogenicity, making them attractive for researchers aiming at developing molecular recognition tools for amyloidogenic proteins. Aptamers typically are obtained by selection from a random-sequence oligonucleotide library based on their affinity for the target of interest using a method called systematic evolution of ligands by exponential enrichment (SELEX, Fig. 2). The oligonucleotides usually span 30 to 100 nucleotides in length and their dissociation constants in complexes with their targets range from pM to mM. The sequence also contains constant regions required for enzymatic manipulation, such as PCR-primer binding and *in vitro* transcription.

**Figure 2:**
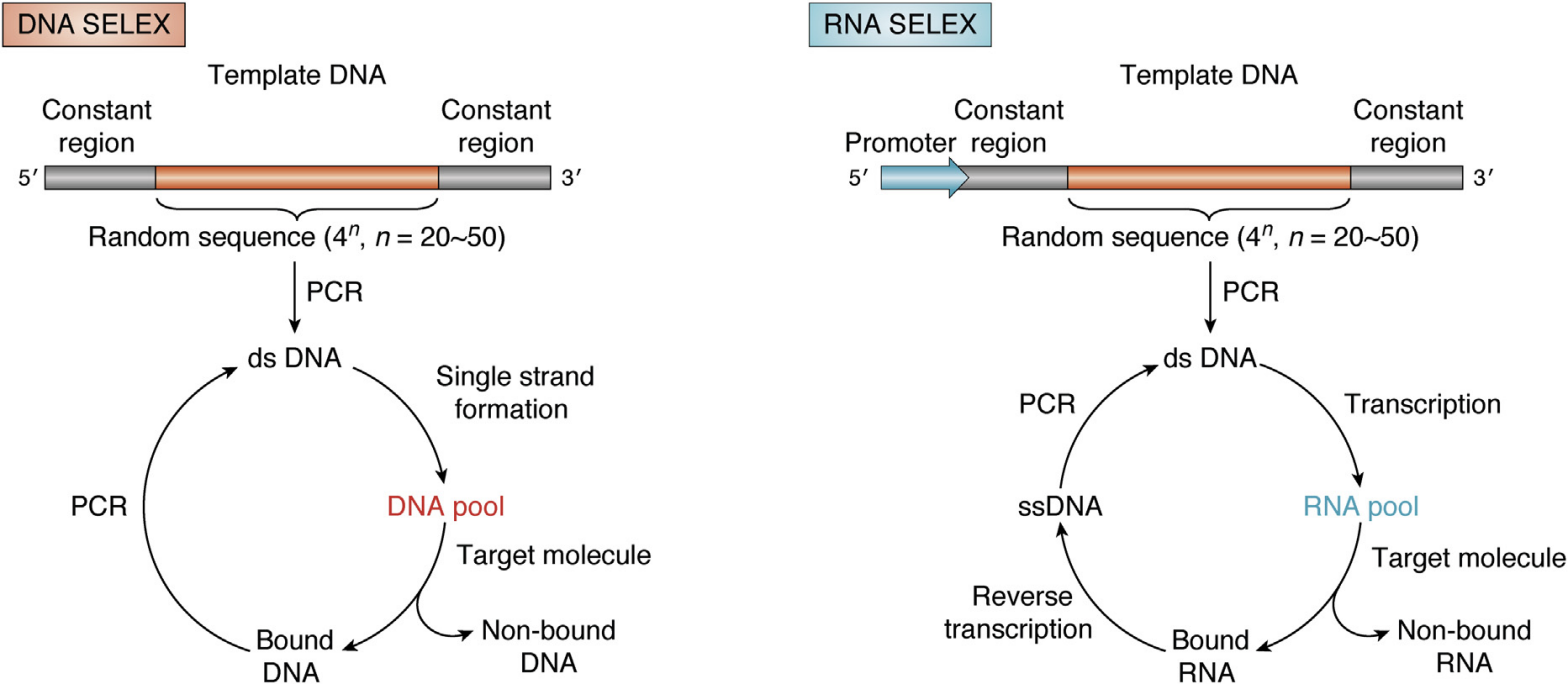
**Generation of aptamers by SELEX.** The process of SELEX can be used for selection of DNA or RNA aptamers. After the initial PCR amplification of the template DNA, single-strand nucleic acid sequences need to be prepared from the double-strand DNA for binding to the target and selection of high-affinity sequences. This is done using NaOH denaturation or enzyme digestion for DNA aptamers and by *in vitro* transcription for RNA aptamers. Unbound sequences are discarded and the bound oligonucleotide pool is released from the target. DNA sequences are subjected to PCR amplification to produce double-strand DNA for the next round of selection, whereas RNA sequences are reverse-transcribed first and then amplified by PCR for the next cycle.

A systematic survey by Dumontier, DeRosa, and their colleagues (9) based on 492 published aptamer-related papers has found that the use of DNA aptamers is increasing compared with RNA aptamers. Each nucleic acid has its own advantages and limitations. DNA oligonucleotides are more stable than their RNA counterparts to enzymatic and chemical degradation and are therefore easier to work with. On the other hand, the presence of the 2′-OH in ribose, as opposed to deoxyribose, and the absence of the 5′-methyl group in uracil compared with thymine allow higher conformational stability of RNA, potentially increasing their affinity for the target (10). Deciding whether to use DNA or RNA aptamers is thus a crucial step in the beginning of every aptamer-based research project.

The application of aptamers in the neurodegenerative-disease field has been reviewed in the past (11, 12, 13), yet to our knowledge, there are no systematic reviews on aptamers targeting amyloidogenic proteins. The metastable nature of intrinsically disordered or misfolded proteins, which are prone to form toxic oligomers and eventually amyloid, makes these proteins one of the most challenging targets for aptamer generation. In the case of oligomers, the aptagens (equivalent to antigens) presented to the oligonucleotide library constantly change, whereas in the amyloid fibrils, the conformation of the aptagens can be both variable for the same protein due to formation of different strains and similar for different proteins in the core cross-β structure shared by most amyloid fibrils.

Aptamers selected against amyloidogenic proteins can have various applications, including sensitive detection of biomarkers, as selective inhibitors of the self-assembly process, and as tools for probing molecular mechanisms. The first application—using aptamers as probes for biomarkers—has been developing rapidly and is too large to include in this review. Therefore, we focus here on the challenges and potential solutions in this field and on preclinical therapeutic applications of aptamers specific for amyloidogenic proteins.

To overcome the difficulty in selecting aptamers against metastable protein assemblies, approaches using stable mimics of these assemblies could be useful if the stabilized molecules represent accurately the metastable target. Here, we provide an update on the development of specific aptamers against amyloidogenic proteins, including amyloid β-protein (Aβ), tau, α-synuclein (αSyn), and prion protein (PrP). These proteins represent a range of sizes spanning an order of magnitude—from 40- to 441-amino acid residues, and the two main mechanisms of initial misfolding and aggregation—partial folding of an unstructured protein, such as Aβ, αSyn, or tau, and partial unfolding of a structured protein—PrP (Fig. 1) (14). We discuss how bioinformatics-assisted approaches or artificial-intelligence-based technologies are used to assist the aptamer selection and optimization processes. We also examine approaches for analysis of the secondary and tertiary structures of aptamers and aptamer–target interaction and strategies for CNS-targeting delivery of aptamers for future development of therapeutics in neurodegenerative diseases.

## Development of aptamers against amyloidogenic proteins

Several dozen amyloidogenic proteins play major deleterious roles in over 50 proteinopathies. However, studies of aptamers against these proteins have concentrated primarily on four proteins, Aβ, tau, αSyn, and PrP, and therefore, these proteins are also the focus of this review.

### Aptamers against Aβ

AD is the most common neurodegenerative disease. Amyloid fibrils in senile plaques in the AD brain consist mainly of the 42-amino acid residue form of Aβ, Aβ42, whereas vascular deposits comprise predominantly the 40-residue form, Aβ40. Both forms are generated from the Aβ-protein precursor by the somewhat promiscuous protease, γ-secretase (15, 16, 17). Aβ42 aggregates faster (18), forms higher-molecular-weight oligomers (19), and is more neurotoxic than Aβ40 (20), and the oligomers of both isoforms are more neurotoxic than the corresponding fibrils (21, 22), making Aβ42 oligomers a primary target for therapy development. In this context, it is important to consider, however, that fibrils might sequester the more toxic Aβ oligomers and possibly are a way cells attempt to reduce the damage caused by the oligomers (23). Thus, if strategies targeting fibril dissociation are considered, inadvertent increase in the concentration of the toxic oligomers, leading to exacerbation, rather than amelioration, of the disease might occur, and one must ensure that this is not the case.

A complicating factor is that “oligomer” is a loosely defined term used for anything from a dimer to large assemblies consisting of hundreds of monomers (22, 24, 25, 26) as long as these assemblies are soluble in aqueous solutions, as opposed to fibrils and other insoluble aggregates. Many different types of Aβ oligomers have been reported, including paranuclei (5,6-mers) (19), Aβ∗56 (56 kDa, 12-mer) (27), protofibrils (PFs, 24–700-mer) (28, 29, 30), globulomers (38/48 kDa, ∼12-mer) (31), AβO (∼90 kDa, 15–20-mer) (32), Aβ-derived diffusible ligands (ADDLs, ∼90 kDa, ∼24-mer) (33), annuli (150–250 kDa, ∼50-mer) (34), and amylospheroids (ASPD; 158–669 kDa, ∼100-mer) (35). Thus, when one contemplates selection of aptamers against Aβ oligomers, an important step is definition of the oligomers used as a target for the selection process.

Protofibrils and ADDLs were the first types of Aβ oligomers described, in 1997 (28, 30) and 1998 (33), respectively. Follow-up studies have generated antibodies against these assemblies, including mAb158 (36) and NU-1 (37), which bind protofibrils and ADDLs, respectively. Many other oligomer-selective antibodies have been reported over the last 2 decades (36, 37, 38, 39, 40, 41) as reviewed elsewhere (25), though due to the metastable nature of the targets, in all cases the specificity of the antibody was lower than what is typically expected for antibodies against stable antigens. Thus, stabilization of the antigens, for example, by attachment of Aβ to gold particles (38), has been an important strategy for generation of antioligomer antibodies. However, an important consideration is that the relevance of the stabilized antigens to the pathological species in the AD brain must be established in each case. Of the many clinical trials using anti-Aβ antibodies, more recent ones have examined antibodies selective against Aβ oligomers, including Biogen’s aducanumab, which recently was approved by the Food and Drug Administration (FDA), and BAN2401, a humanized version of mAb158.

The first aptamers targeting Aβ were reported in 2002 by Ylera *et al.* The aptamers were RNA oligonucleotides screened against Aβ40 monomers tethered to a Sepharose support. All the selected aptamers recognized Aβ fibrils but not monomers (42). In a later study by Hyman and coworkers (43), one of the aptamers reported by Ylera *et al.*, β55, was used to visualize senile plaques using multiphoton microscopy in brain tissue from patients with AD and the APP/PS1 mouse model (44). The characteristics of these anti-Aβ aptamers and all the subsequent aptamers against amyloidogenic proteins included in this review are summarized in Table 1.

**Table 1:** Characteristics of aptamers against Aβ, tau, αSyn, and PrP

Name	Target	Nucleic acid	*K*_D_ (testing method)	Selectivity	Year	Ref.
Anti-Aβ aptamers						
β55	Aβ40 monomers	RNA	29 nM (affinity chromatography)	fibrils	2002	(42)
KM33	Aβ40 trimers	RNA	N.T.^a^	fibrils	2009	(48)
T-SO508	αSyn oligomers	DNA	25 nM for Aβ40 oligomers (ELONA^b^)	oligomers^c^ of αSyn and Aβ40	2012	(51)
E2	Aβ40	RNA	10.9 μM (fluorescence anisotropy)	fibrils	2009	(53)
RNV95	Aβ40	DNA	N.T.	oligomers (∼75 kDa and ∼150 kDa)	2018	(55)
E22P-AbD43	Aβ42 protofibrils	RNA	20 nM (BLI^d^)	dimers	2020	(58)
Aβ7-92-1H1	Aβ42	DNA	53.3 nM (SPR^e^)	oligomers	2020	(64)
Antitau aptamers						
ssDNA_1_	1N3R-tau	DNA	190 nM (capillary electrophoresis)	monomers	2005	(66)
3146	2N4R-tau	DNA	13 nM (SPR)	monomers	2018	(69)
tau-1	2N4R-tau	RNA	N.T.	dimers and trimers	2016	(70)
Anti-αSyn aptamers						
M5-15	αSyn	DNA	N.T.	monomer and oligomers	2010	(80)
T-SO530	αSyn oligomers	DNA	63 nM for αSyn oligomers (ELONA)	oligomers of αSyn and Aβ40	2012	(51)
F5R1	αSyn	DNA	2.4 nM (SPR)	N.T.	2018	(82)
Antiprion aptamers						
Ap1	hamster PrP23–231	RNA	N.T.	hamster, mouse, cattle PrP^C^	1997	(93)
60–3	mouse PrP23–230	RNA	5.6 nM for PrP^C^ (competitive assay)	mouse and bovine PrP^C^	2006	(94)
RM312	sheep PrP23–234	RNA	15 nM for PrP^C^ (SPR)	N.T.	2006	(95)
4–9	mouse PrP23–230	DNA	113 nM for PrP^C^, 100 nM for PrP^Sc^ (SPR)	PrP^C^ ≅ β-isoform of PrP^Sc^	2007	(96)
4C26	mouse PrP90–231	DNA	18 nM for PrP^C^ (affinity assay)	PrP^C^ (N.T. for PrP^Sc^)	2008	(97)
SAF-93	PrP fibris from infected hamster brain	RNA	23.4 nM for PrP^Sc^ (affinity assay)	PrP^Sc^ > PrP^C^	2003	(98)
DP7	human PrP90–129	RNA	1.7 μM for PrP^C^ (affinity assay)	human, hamster, mouse PrP^C^	2002	(100)
SSAP3-10	human PrP23–231	DNA	N.T.	human, sheep, calf, piglet, deer PrP^C^, but not PrP^Sc^	2006	(101)
R14 (from apt#1)	bovine PrP25–241	RNA	8.5 nM for PrP^C^, 280 nM for PrP^Sc^ (affinity assay)	PrP^C^ > PrP^Sc^	2008	(102)

a Not tested.

b Enzyme-linked oligonucleotide assay.

c Size is unspecified, unless stated otherwise.

d BioLayer interferometry.

e Surface plasmon resonance.

**Figure 3:**
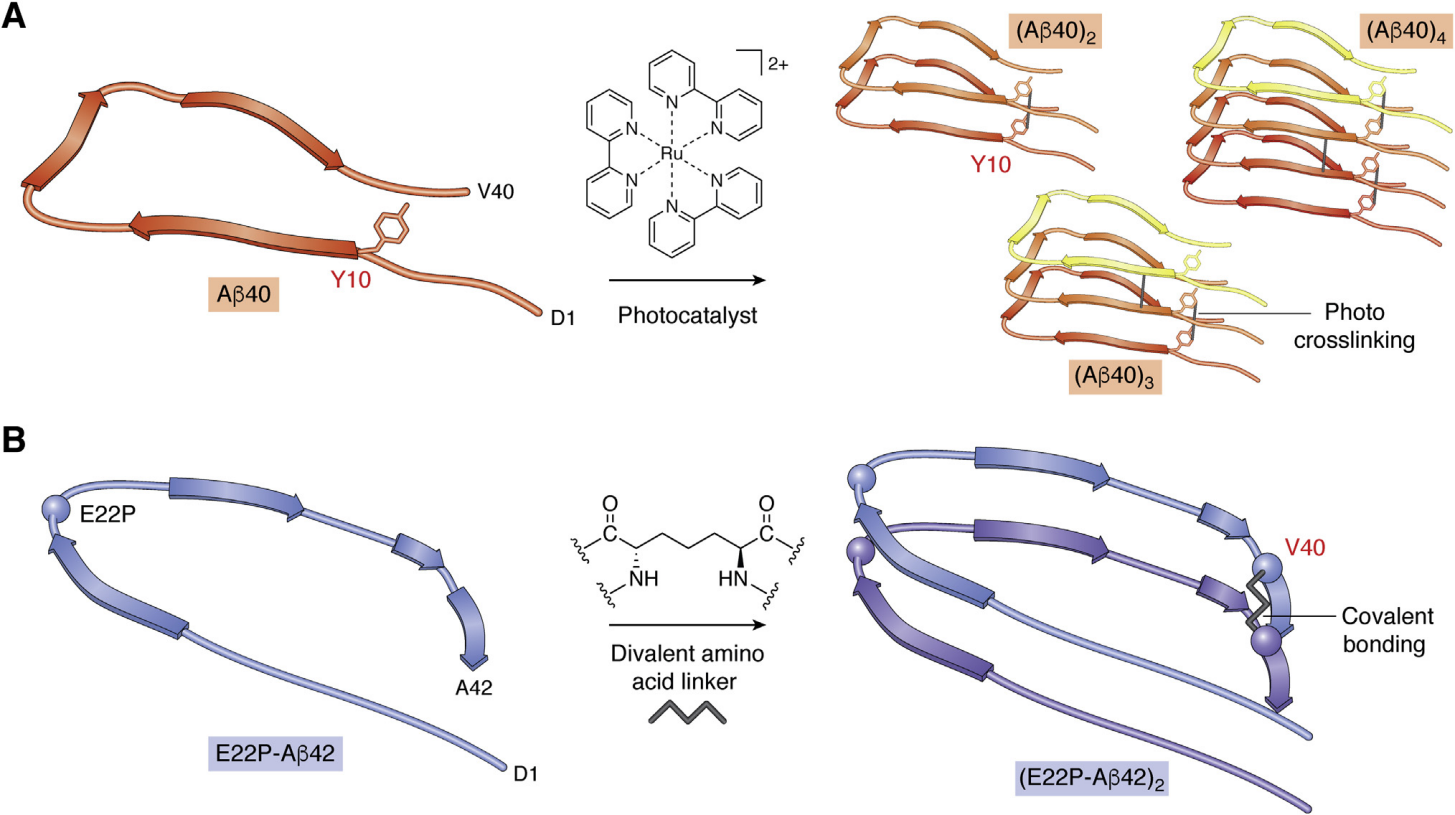
**Strategies for stabilization of Aβ oligomers for generation of aptamers.***A*, photo-cross-linking of Aβ40 by PICUP. Cross-linking is induced by visible-light irradiation in the presence of the photocatalyst tris(bipyridyl)ruthenium(II) ([Ru(bpy)_3_]^2+^) and the electron acceptor ammonium persulfate. This method leads to “zero-length” cross-linking directly between amino acid residues, primarily tryptophane and tyrosine. In Aβ, the main cross-link is at Y10, though other bonds also can form. *B*, a turn structure in Aβ42 is stabilized by an E22P substitution. Dimer stabilization is achieved through substituting V40 by a divalent amino acid (*e.g.*, 2,6-diaminopimelic acid).

To select aptamers against Aβ oligomers, several chemical tethering approaches have been used in later studies. Rahimi *et al.* prepared covalently cross-linked Aβ40 oligomers using Photo-Induced Cross-linking of Unmodified Proteins (PICUP) (45, 46), which creates covalent bonds at unspecified positions, primarily at Tyr10 in Aβ (Fig. 3*A*) (47). Of several Aβ40 oligomers stabilized by this technique, they then isolated the most abundant type, trimers, using SDS-PAGE and used the cross-linked trimers to isolate RNA aptamers out of a 77-nucleotide library including 49 randomized nucleotides (A:U:G:C at equal ratios). This size library contains 3.2 × 10^29^ unique sequences theoretically, yet in reality, if every sequence indeed was included in the library, the mass of such a library would be too high, and the actual number of unique sequences is lower.

Although the selection of the RNA aptamers was against Aβ40 trimers, the final aptamers, KM33 and KM41, were not selective for trimers or other oligomers, but bound to Aβ40 fibrils and did not display a higher affinity for the fibrils than the naive oligonucleotide library used for the selection before enrichment (48, 49). Part of the explanation of these results was that the PICUP-immobilized Aβ40 trimers themselves might have aggregated during the selection process to form fibrillar structures. Further analysis suggested that oligonucleotides have a high, nonspecific affinity for amyloid fibrils, as KM33 and KM41 also recognized fibrils of other amyloidogenic proteins, including calcitonin, islet amyloid polypeptide, insulin, lysozyme, and PrP106–126 (48), which share a cross-β structure and fibrillar morphology with Aβ fibrils (50). Thus, Rahimi *et al.* (48) demonstrated that the aptamers could be used to monitor fibril formation in a similar manner to thioflavin T (ThT) fluorescence.

Interestingly, a DNA aptamer called T-SO517, originally developed by Ikebukuro and colleagues against oligomeric αSyn, a key protein involved in PD and other synucleinopathies, bound not only to their intended target, αSyn oligomers, but also to Aβ40 oligomers (51), demonstrating the difficulty in obtaining highly specific aptamers against assemblies of amyloidogenic proteins. Considering the high affinity of the KM aptamers mentioned above for fibrils, these findings likely reflect the presence of common structures in such assemblies, *i.e.*, not only the common cross-β structure of the fibrils, but also in the oligomers, as has been suggested by binding of antioligomer antibodies, such as A11, to oligomers of multiple amyloidogenic proteins (38). In an attempt to develop a detection system for Aβ40 oligomers, T-SO517 was applied to a fluorescence detection system using abasic site-containing DNA oligonucleotides (52). When monitoring the Aβ40 aggregation process, this system showed preference for detecting oligomers over monomers or fibrils *in vitro*, which was confirmed by transmission electron microscopy.

Using a different selection approach, Takahashi *et al.* (53) tethered Aβ40 to colloidal gold nanoparticles as a target for selection of RNA aptamers using the same method previously developed by Kayed *et al.* (38) for preparation of oligomer-specific antibodies. The RNA aptamers, N2 and E2, bound to Aβ40 monomers and inhibited Aβ40 fibril formation assessed using the ThT fluorescence assay and transmission electron micrography. However, as was found by Rahimi *et al.* (48), Babu *et al.* (54) demonstrated later by using atomic force microscopy that these aptamers also bound to Aβ40 fibrils. Binding to Aβ- or other protein oligomers was not described in either study. A DNA aptamer named RNV95, selected using column-immobilized Aβ40 by Chakravarthy *et al.* with the goal of detecting low-molecular-weight Aβ40, recognized human brain Aβ oligomers at both ∼75 kDa and ∼150 kDa. However, it was not tested for binding to Aβ fibrils or fibrils of other amyloidogenic proteins so its selectivity for oligomers was not established (55).

To our knowledge, to date, only two studies have explored development of aptamers against Aβ42, likely because it is an even more difficult target than Aβ40. Aβ42 has been shown to aggregate faster (18), form distinct oligomers (19, 56), and cause stronger neurotoxicity than Aβ40 (21, 57). The first characteristic, substantially faster aggregation, makes working with Aβ42 particularly difficult because the preparation of the protein changes during the experiment making obtaining reproducible data highly challenging.

The first example of a successful generation of aptamers against Aβ oligomers we are aware of is by Murakami *et al.*, who developed RNA aptamers termed E22P-AbD4, -AbD31, and -AbD43 against Aβ42 protofibrils (58, 59). To select the aptamers, a dimer of E22P-Aβ42 was used in which the monomers were tethered covalently by a bivalent amino acid linker in place of Val40, within the C-terminal hydrophobic region of Aβ42 (Fig. 3*B*) (60). Upon incubation in phosphate buffer at 37 °C without agitation for 48 h, this dimer construct, in which a turn near Glu22 was stabilized by an E22P substitution (61), formed protofibrils, the morphology of which was confirmed by transmission electron microscopy. The protofibrils then were used in SELEX to obtain the aptamers from a 77-nucleotide RNA library including a 49-nucleutide random sequence. When used in histological experiments, all three aptamers stained diffuse oligomeric aggregates in two mouse models of AD, Tg2576/PS2 (62) and *App*^NL-G-F/NL-G-F^ (63), suggesting that the Aβ assemblies formed in the brain of these two mouse models contained similar protofibril-derived aptatopes. Incubation of the E22P-Aβ42 dimer in the presence of aptamer E22P-AbD43 showed that the aptamer inhibited the nucleation phase of the protofibril formation. The aptamer also inhibited dose-dependently the neurotoxicity of both the E22P-Aβ42 dimer and Aβ42 in the neuroblastoma cell line SH-SY5Y cells. Computational and two-dimensional structure analysis of E22P-AbD43 suggested that preferential binding of the aptamer to Aβ42 protofibrils compared with fibrils might be related to formation of a G-quadruplex structure, implying the presence of a common structure in protofibrils made of either the synthetic dimer or native Aβ42 protofibrils (59).

For the purpose of developing inhibitors of Aβ42 aggregation, Zheng *et al.* selected an anti-Aβ42 DNA aptamer (Aβ7-92-1H1) by incubating a library of DNA-oligonucleotide-coated beads with nonaggregated Aβ42. This library consisted of 100-nucleotide-long sequences including two separate 18-nucleotide random sequences. Aβ7-92-1H1 bound Aβ42 and neither Aβ40 nor other amyloids. The affinity of the aptamer for Aβ42 oligomers was slightly higher than for Aβ42 monomers, measured by surface plasmon resonance (SPR). The aptamer inhibited Aβ42 fibril formation as evidenced by atomic force microscopy and β-sheet formation measured using CD spectra (64).

### Aptamers against tau protein

Tauopathies are neurodegenerative diseases caused by formation of toxic oligomers and aggregates of the microtubule-associated protein tau. Tau is produced as six different isoforms in humans, due to alternative splicing of exons 2, 3, and 10 leading to polypeptides ranging from 352 to 441 amino acid residues. Translation of neither, one, or both exons 2 and 3 is marked as 0N, 1N, or 2N isoforms of tau, whereas the alternative splicing of exon 10, which encodes part of the microtubule binding, repeat domain of tau leads to isoforms containing three (3R) or four (4R) repeats.

The most prevalent tauopathy is AD (65). Other examples of tauopathies include frontotemporal lobar degeneration, progressive supranuclear palsy, and chronic traumatic encephalopathy. Aggregation and deposition of tau, *e.g.*, as neurofibrillary tangles in AD, are associated with hyperphosphorylation and other aberrant posttranslational modifications of the protein.

In the course of testing whether tau binds single-strand DNA, Krylova *et al.* (66) first found DNA sequences binding recombinant 1N3R-tau or 2N3R-tau using nonequilibrium capillary electrophoresis of equilibrium mixtures, in which the gel shift caused by binding of the DNA oligonucleotides to tau was evaluated. In follow-up studies, these DNA sequences were used as aptamers for detection of 1N3R-tau in human plasma using SPR and were shown to reach femtomolar level sensitivity (67). Selection of aptamers without amplification between selection rounds shortens the time and saves costs compared with conventional SELEX (68). However, this is not a standard method in the amyloid field. Lisi *et al.* applied this method coupled with capillary electrophoresis for partitioning of bound DNA from unbound DNA to isolate DNA aptamers targeting several tau isoforms. The aptamer 3146 was obtained from a 5 × 10^12^ DNA oligonucleotide library in only three rounds within 1 day. 3146 bound tau isoforms in the order 2N4R (*K*_D_ = 13 nM) > 0N4R (49 nM) > 0N3R (84 nM) > 1N3R (116 nM) (69).

An RNA aptamer called tau-1 was obtained against 2N4R-tau, by applying SELEX to a 90-nucleotide RNA library containing a 40-nucleotide random region. This aptamer prevented the formation of 2N4R-tau dimers and trimers assessed by SDS-PAGE/western blotting (70). It is important to note, however, that SDS-PAGE is not a reliable method for assessing oligomer formation by amyloidogenic proteins, as SDS perturbs protein conformation and can both dissociate existing assemblies and induce formation of different assemblies (26). Nonetheless, in an experiment using a HEK293-derived cell line expressing human 2N4R-tau under control of doxycycline induction (71), reduced cell viability resulting from tau expression was recovered by treating with tau-1 compared to a random RNA library (70).

### Aptamers against α-synuclein

αSyn, a causative agent of synucleinopathies, such as PD, dementia with Lewy bodies, and multiple system atrophy, oligomerizes and aggregates in the brain of patients leading to neurotoxicity and neurodegeneration (72). αSyn is a 140-amino-acid long protein mainly located at presynaptic terminals. The amphipathic N-terminal region (residues 1–60), which includes four 11-residue imperfect repeats, and the hydrophobic middle region (residues 61–95) are more important for aggregation than the acidic and proline-rich C-terminal region (residues 96–140) of αSyn (73). The C-terminal region of αSyn is susceptible to cleavage upon aggregation (74), yet most antibodies bind to this region and therefore may miss αSyn aggregates in pathological analysis of patient brains or animal models. This problem is particularly important because the most common form of αSyn used to detect pathological aggregates is phosphorylated at Ser129 (pS129-αSyn), yet some cleavage sites are N-terminal to position 129 and eliminate the epitope for antibodies against pS129-αSyn.

Similar to Aβ and tau, αSyn oligomers, rather than fibrils, are thought to be the primary neurotoxic form of the protein. αSyn oligomers cause neurotoxicity, synaptic impairment, mitochondrial dysfunction, endoplasmic reticulum stress, neuroinflammation, proteostasis dysregulation, and apoptosis, culminating in neuronal death (75). Recent cryo-electron microscopy analyses have deciphered the atomic structure of αSyn filaments (76) and structure–activity analysis suggested that the N-terminus controls αSyn aggregation (77). The details of αSyn oligomer structures are not known, yet β-sheet structure has been reported in rigid regions of toxic αSyn oligomers, whereas in nontoxic oligomers, these regions are unstructured (78). The mechanism underlying oligomer toxicity may involve insertion into lipid bilayers, disrupting membrane integrity (78). Bioinformatic and NMR studies have supported an important role for the N-terminus in modulating the aggregation of αSyn (79).

**Figure 4:**
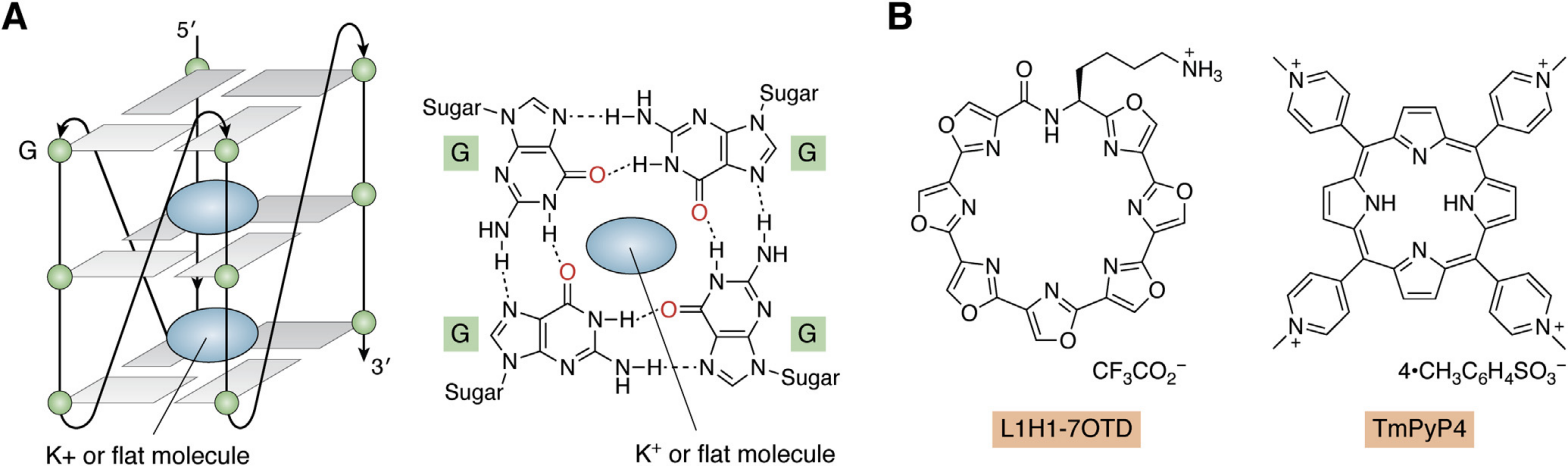
**G-quadruplex structures in aptamers and their stabilization.***A*, a schematic structure of a G-quadruplex, which can be stabilized by a metal ion, *e.g.*, K^+^, or flat molecules, for example those shown in panel *B*. *B*, structures of L1H1-7OTD and TmPyP4, which have been used as stabilizers of a G-quadruplex in the anti-αSyn aptamer T-SO530.

Ikebukuro and colleagues reported a first DNA aptamer against αSyn, called M5-15, which showed affinity for both monomers and oligomers (80). In follow-up studies, the same group applied counterselection of monomers and fibrils, leading to a more selective aptamer, T-SO530, against αSyn oligomers, whose selectivity was confirmed by dot blots (51). CD analysis suggested that T-SO530 formed a G-quadruplex structure, which might have contributed to its affinity. Indeed, G-quadruplex stabilizers, such as L1H1-7OTD and TmPyP4 (Fig. 4), enhanced the binding of T-SO530 to αSyn oligomers (81), suggesting that such stabilizers can be promising synthetic modulators/cofactors for applications of aptamers against amyloidogenic proteins in neurodegenerative diseases.

Zheng *et al.* (82) developed 58-nucleotide DNA aptamers they termed F5R1 and F5R2, starting with a 40-nucleotide random single-strand DNA library, which bound αSyn with a high affinity and inhibited αSyn aggregation. To enhance cell membrane permeability of the aptamers, they were modified by attachment of a peptide carrier, CADY, which had been reported previously to form stable complexes with nucleic acids, leading to improvement of their delivery into cultured cells (83). The modified aptamers reduced intracellular aggregation of αSyn, synaptic protein loss, and neuronal death caused by αSyn overexpression.

### Aptamers against prion protein

The accumulation of misfolded PrP characterizes prionoses, such as Creutzfeldt-Jakob disease, Kuru, Gerstmann–Sträussler–Scheinker Syndrome, and fatal familial insomnia in humans, bovine spongiform encephalopathy in cattle, scrapie in sheep, and chronic wasting disease in deer and elk (84, 85, 86). PrP exists normally as PrP^C^ (cellular form), which is involved in neuroprotection and trophic signaling (87, 88, 89), yet this form can misfold due to genetic, environmental, or yet unknown causes into a toxic and infectious form called PrP^Sc^ (scrapie form). The term PrP^Sc^ does not describe one particular structure but refers to many conformational strains that are self-propagating, transmissible from cell to cell, and are infectious within the same species and sometimes across species. PrP^Sc^ infectivity to other organisms depends on the specific strain and species barrier. PrP^C^ is rich in α-helix, whereas PrP^Sc^ contains the typical cross-β structure of amyloid fibrils. The aggregation process, including oligomerization of PrP^Sc^, plays a central role in the prion’s propagation and neurotoxicity (90). Biochemically, PrP^Sc^ is a highly stable form of the protein, which is resistant to proteinases and denaturing agents.

Interestingly, one of the biological roles of PrP^C^ is a receptor of Aβ oligomers. Binding of Aβ oligomers to PrP^C^ causes synaptotoxicity and neuritic dystrophy, possibly also leading to tauopathy (91, 92). Thus, both PrP^C^ and PrP^Sc^ are potential therapeutic targets for development of various drug modalities, including aptamers.

Weiss *et al.* reported the first RNA aptamer, Ap1, against a recombinant Syrian golden hamster prion protein PrP23–231, a common model of full-length PrP^C^. The aptamer recognized PrP^C^ in brain extracts of scrapie-infected mice, hamsters, and cattle, though its binding affinity was not measured (93). An RNA aptamer called “60-3”, selected against mouse PrP23–230, was reported by Sekiya *et al.* (94) and also bound bovine PrP25–241. Mercey *et al.* (95) developed an RNA aptamer, RM312, against sheep PrP23–234, which bound with 15 nM affinity to PrP^C^. In another study, Ikebukuro and colleagues developed a DNA aptamer, termed “4-9”, against mouse PrP23–230 that bound to both PrP^C^ and PrP^Sc^ equally (96). A DNA aptamer called 4C26 was prepared by Garson and colleagues against mouse PrP90–231 and exhibited a higher binding affinity for mouse prion than “4-9” (97) suggesting that shortening the target length in this case improved the affinity.

To pursue aptamers selective for PrP^Sc^ *versus* PrP^C^, an RNA aptamer, SAF-93, was developed against scrapie-associated fibrils from the brains of infected hamsters. In a competitive binding assay using bovine PrP^Sc^ fibrils, SAF-93 showed >10-fold higher affinity for PrP^Sc^ than for PrP^C^ (98). Uniquely, SAF-93 was obtained by SELEX using 2′-fluoro-modified pyrimidine triphosphate nucleotides together with unmodified purine nucleotides. Enzymatic probing and gel footprinting, in addition to computer-assisted secondary structure analysis, identified multiple binding sites of SAF-93 on PrP^Sc^ (99).

Several aptamers also have been selected against human prion proteins. An RNA aptamer termed DP7 was prepared by Proske *et al.* against human PrP90–129, an important domain for the conversion of PrP^C^ into PrP^Sc^. The aptamer also bound PrP derived from hamster and mouse. The utility of this aptamer for reducing the ratio of PrP^Sc^ to PrP^C^ was demonstrated in prion-infected mouse neuroblastoma N2a cells (100). Takemura *et al.* (101) reported a DNA aptamer called SSAP3-10 against human PrP23–231, which bound other mammalian PrP^C^ (sheep, calf, piglet, and deer) in addition to human PrP^C^, but not to PrP^Sc^.

**Figure 5:**
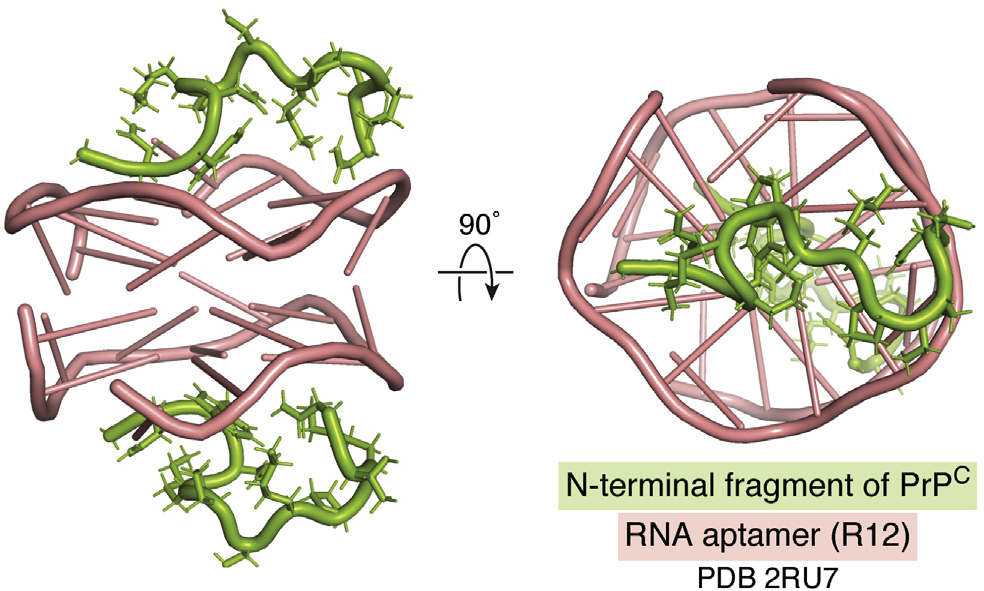
**Stabilization of a complex between the antiprion RNA aptamer R12 and the N-terminal region of PrP**^**C**^**.** A dimer of R12 [*pink*, r(GGAGGAGGAGGA)] associates with two N-terminal PrP^C^ peptides (*green*, Gly-Gln-Trp-Asn-Lys-Pro-Ser-Lys-Pro-Lys-Thr-Asn) providing a 1:1 stoichiometric ratio. The illustration was created using PyMOL from PDB ID: 2RU7.

Structural studies of prion aptamers have been advanced compared with other amyloidogenic proteins. Murakami *et al.* developed an RNA aptamer, apt#1, selected against bovine PrP25–241. The binding was deduced to be due to a G-quadruplex structure using circular dichroism (CD) spectroscopy. An extracted sequence from apt#1 (R14 aptamer, GGAGGUUUUGGAGG) was identified using mutagenesis and showed improved binding affinity and ∼30-fold selectivity for PrP^C^ compared with PrP^Sc^ (102). Katahira and colleagues further optimized the sequence to deduce a tandem repeat sequence (R12 aptamer, (GGA)_4_), and found using NMR measurements that it formed an intramolecular parallel G-quadruplex (103). R12 bound to two sites in the N-terminal half of PrP^C^ by forming a dimer (104, 105) (Fig. 5). Treatment with R12 reduced the level of PrP^C^ (104) and blocked the pathological conformational conversion of PrP^C^ into PrP^Sc^ in scrapie-infected mouse neuronal GT1-7 cells (106).

## Computer-assisted development and structure prediction of aptamers

### Selection and clustering of aptamers by high-throughput sequencing

Despite the large potential of aptamers as therapeutic agents, biosensors, and research tools, technical issues can require major time and money investments, limiting development and progress. One such technical issue is the use of classic Sanger sequencing of the selected oligonucleotides. Although the selection process removes the vast majority of the sequences, the final pool still may contain thousands of distinct oligonucleotide sequences, making sequencing of this pool and identifying the sequences possessing the best affinity and/or specificity labor-intensive and time-consuming.

*In silico* approaches, previously reviewed by Hamada (107), have been useful for aptamer design. Artificial intelligence (AI) coupled with machine learning algorithms assists in identifying potential aptamer candidates from the selected sequences expeditiously, leading to improvement compared with classical prediction tools (108). As only the oligonucleotide pool of the last round of selection typically is cloned and sequenced in conventional SELEX, superior aptamers may be overlooked. In this section, we discuss the role of bioinformatics in aptamer development, including in sequencing, secondary-structure prediction, and simulation of aptamer–target interaction, and highlight the use of these advances in selection of aptamers against amyloidogenic proteins.

With the advent of new sequencing technologies, such as Next-Generation sequencing, exhaustive parallel sequencing of all the selection rounds has facilitated high-throughput SELEX (HT-SELEX) (109, 110, 111). In the first report of this technique, a *K*_D_ in the picomolar range was achieved after only three rounds of selection for aptamers against the BB subunits of platelet-derived growth factor (109). The abundance of sequences read in all the SELEX pools can be ranked by various parameters in HT-SELEX, *e.g.*, sequence enrichment (112), and meta-Z-score (113). The meta-Z-score is a statistical scoring method for high prediction accuracy of binding potential. This method allows gleaning insight into whether sequence enrichment occurs, rather than whether the affinity for the target is enhanced at substantially earlier stages of the aptamer-selection process, resulting in considerable time saving. Additionally, a decreased number of selection rounds helps reduce PCR bias (nonspecific amplification) caused by over selection or excessive cycle numbers in PCR (114). Comprehensive analysis of very large sequence datasets by such bioinformatics methods has enabled not only accurate characterization of aptamers, data alignment, and clustering, but also improved prediction of aptamer structure and aptamer–target interaction mode.

After collecting and ranking datasets of sequences, data clustering typically is a next step, which can be achieved using free software tools, such as AptaCluster (115) (https://www.ncbi.nlm.nih.gov/CBBresearch/Przytycka/index.cgi#aptatools) and FASTAptamer (116) (https://burkelab.missouri.edu/fastaptamer.html), both of which use RNA sequence, but not structural information. Ikebukuro and colleagues proposed a genetic methodology named evolution-mimicking algorithm, to identify optimized aptamer sequences in rugged sequence spaces. Using this methodology, by combining several *in vitro* assays, *e.g.*, binding affinity, inhibitory activity, specificity, and 3D structure, *in silico* maturation that included selection and duplication, recombination, and point mutation has facilitated identifying several aptamers against PrP and αSyn (117, 118, 119).

### Analysis of secondary and tertiary structure of aptamers by motif finding and structure optimization

**Figure 6:**
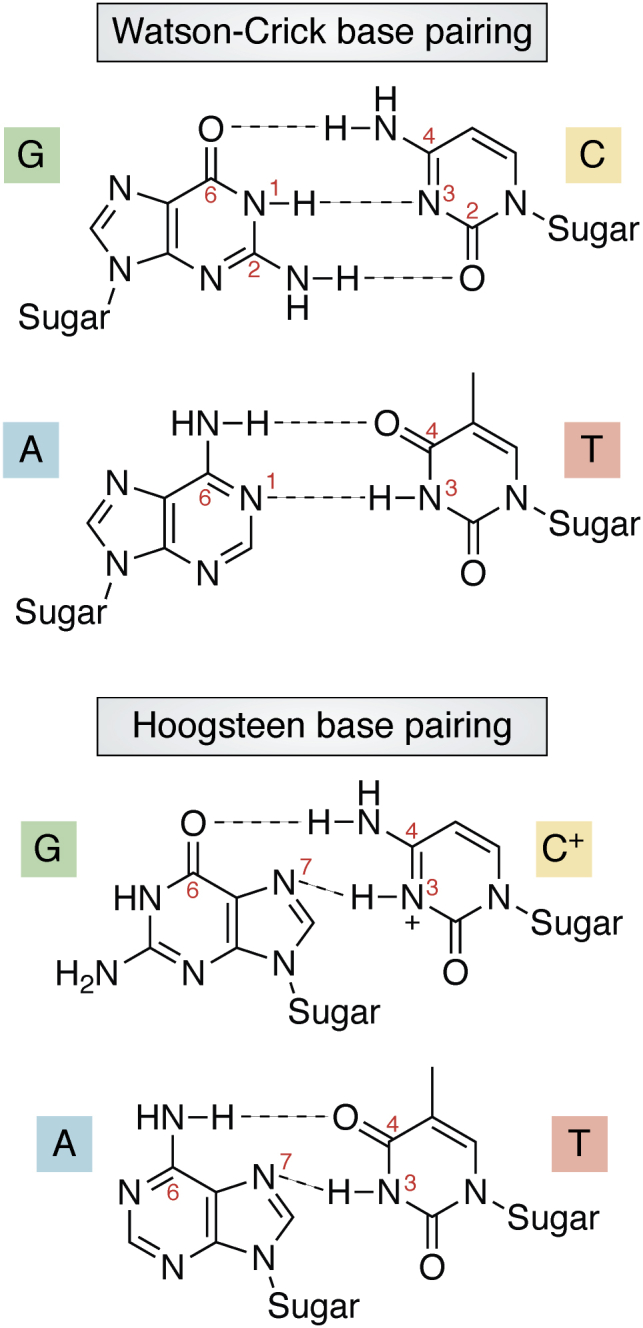
**Canonical and noncanonical stabilization of natural base-pairs.** Canonical, Watson–Crick interactions have three hydrogen bonds between guanine and cytosine (O6–N4, N1–N3, and N2–O2), and two hydrogen bonds between adenine and thymine (N6–O4 and N1–N3). In noncanonical Hoogsteen interactions, the adenine or guanine is rotated 180° around the glycosidic bond, resulting in alternative hydrogen base pairs—two hydrogen bonds between guanine and cytosine (O6–N4 and N7–N3), and two hydrogen bonds between adenine and thymine (N6–O4 and N7–N3). Hoogsteen interactions are minor compared with the Watson–Crick structures but may contribute to formation of unique DNA or RNA structures.

When conducting an aptamer discovery campaign, the three-dimensional structures of the selected aptamers and aptamer–target complexes are important considerations. The relatively simpler conformational space of nucleic acids compared with proteins makes computer-aided calculation and modeling of secondary and tertiary structures particularly useful for aptamers. The secondary structure of oligonucleic acids is determined by canonical Watson–Crick base-paring interactions or noncanonical Hoogsteen base pairs in single or double strands (Fig. 6). Due to the additional hydroxyl group in ribose compared with deoxyribose, RNA single strands are believed to form more complex and diverse structures than DNA, such as stem loops and pseudoknots.

**Figure 7:**
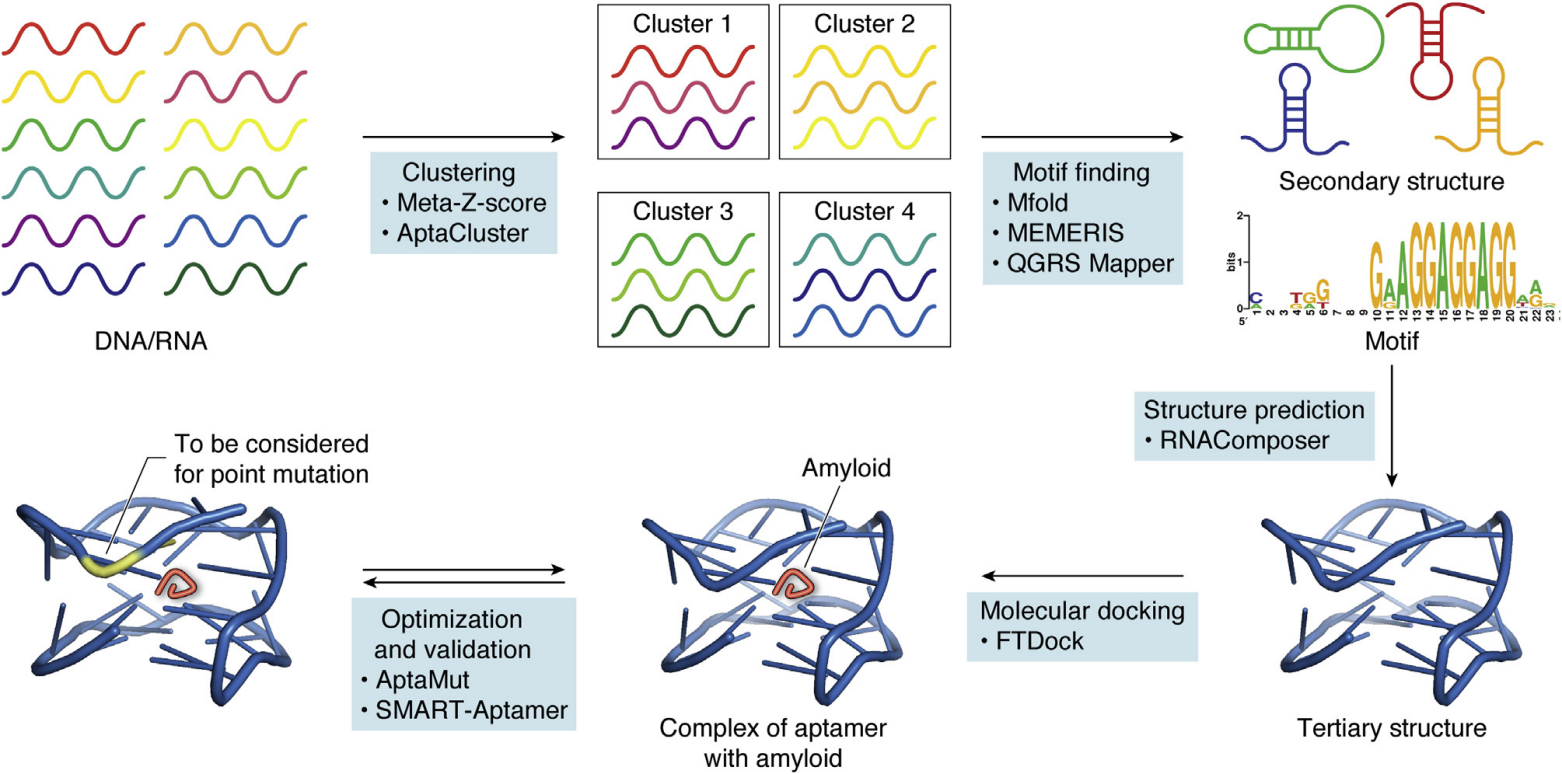
**A recommended workflow for *in silico* aptamer design, analysis, and optimization.** Candidates of DNA or RNA aptamers are subjected to clustering by high-throughput sequence analysis programs, such as meta-Z-score or AptaCluster. To predict secondary structures and motifs, the resulting clusters are processed by secondary-structure and motif-finding software tools such as Mfold, MEMERIS, and/or QGRS Mapper. Next, prediction of the tertiary structure of the aptamers is performed by RNAComposer. Then, the binding site(s) may be deciphered by molecular docking of the aptamers with their target, *e.g.*, an amyloid protein, using machine learning tools, such as FTDock. Finally, the resulting complex structure is further tested by SMART-Aptamer, followed by AptaMut analysis for considering point mutations that may improve the aptamer’s affinity and/or specificity.

The program Mfold, originally developed by Zuker (120, 121) and later modified by incorporating into the University of Wisconsin Genetics Computer Group software suites (122), uses minimal free energy optimization and is one of the most popular tools for determination of nucleic acids’ secondary structure. Typically used after Mfold, MEMERIS is a general motif-finding algorithm for integration of secondary structures (123). AptaMotif is a program designed for identifying binding motifs in aptamers identified through SELEX and is based on structural processing, including suboptimal secondary structures, for prediction of sequence motifs in the loop regions (124). The tertiary structures of aptamers can be predicted based on the calculated information of secondary structures. Online programs and servers are available for this purpose, including RNAComposer (125) and SimRNAweb (126). A putative flow from an identified sequence to computer-aided structural optimization of aptamers is shown in Figure 7. To allow convenient selection of the appropriate tool for each task, Rtools (http://rtools.cbrc.jp/) was developed as an integrated web-server hosting existing prediction algorithms for RNA secondary structure analysis (127).

Structural prediction programs have begun being applied in the amyloid field. Mfold likely is most used program in the field due to its free availability. Mfold analysis of the anti-Aβ40 DNA aptamer RNV95 suggested a stem-loop structure, which was validated by CD spectroscopy measurements (55). Bunka *et al.* (128) utilized Mfold for the prediction of RNA aptamer structure against β_2_-microglobulin, a protein whose deposition as amyloid in joints is associated with symptoms of dialysis-related amyloidosis, to reveal an enzyme cleavage site in a stem-loop structure, facilitating the identification of the aptamer’s aptatope.

G-quadruplex is a noncanonical structure in both RNA and DNA aptamers, which, as mentioned in previous sections, has been found in several cases to play key roles in aptamer binding to amyloid–protein targets. The G-quadruplex structure includes a stable planar core comprising four guanine bases in the same plane forming G-tetrads (Fig. 4*A*) stabilized by π–π interactions (129, 130). The structure can be stabilized further by metal ions (131) or by flat molecule-induced chelation (Fig. 4*B*) (81). However, prediction of G-quadruplex motifs is particularly difficult because of the need to bring together four noncontiguous guanines that may be far apart from each other in the sequence (132).

Several software tools have been developed to address this challenge, such as QGRS (Quadruplex forming G-Rich Sequences) Mapper (133), GRSdb (134), and QuadBase2 (135), which are specific to prediction of G-quadruplexes in RNA and DNA sequences. For example, the formation of G-quadruplex in an RNA aptamer against Aβ42 protofibrils predicted by QGRS Mapper was validated by detection of a negative peak at ∼240 nm and a positive peak at ∼265 nm in the CD spectrum (136), and by observation of a 1650 cm^−1^ absorbance peak in the Attenuated Total Reflection-FTIR spectrum (137), both of which are unique to guanine carbonyl groups in a G-quadruplex (58, 59). Similarly, a DNA aptamer (T-SO508) against αSyn oligomers was predicted to contain a G-quadruplex by QGRS Mapper, and the prediction was confirmed by CD spectroscopy (81).

**Figure 8:**
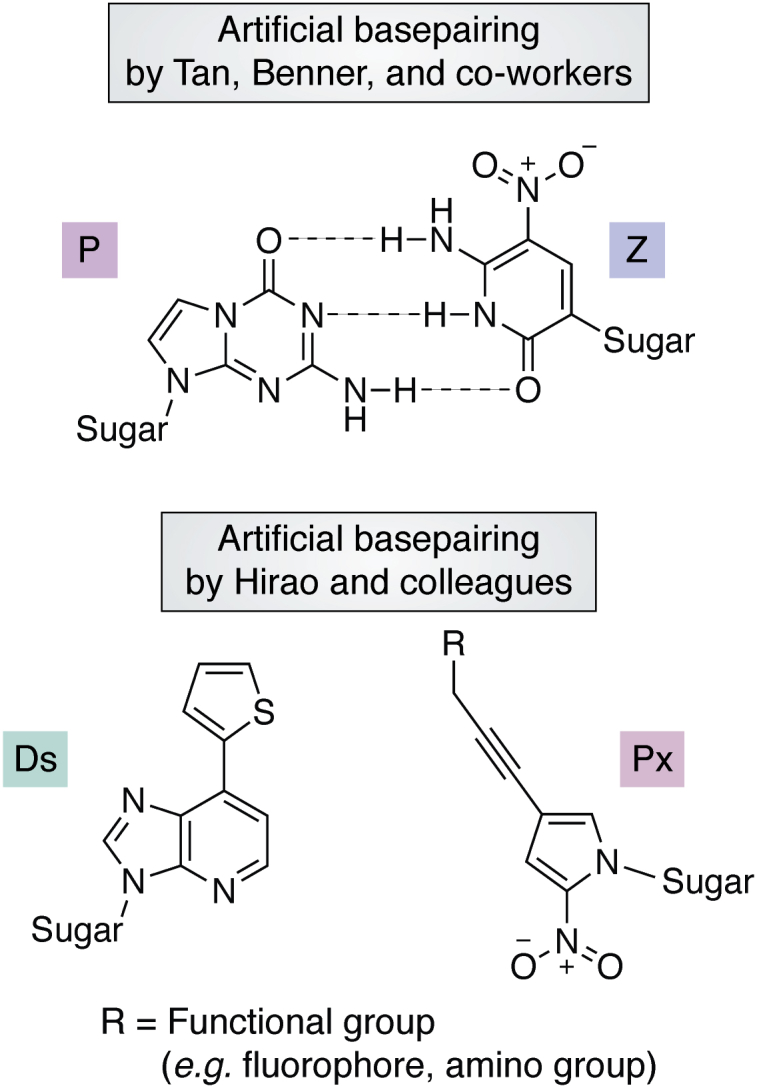
**Noncanonical artificial base pairs used in aptamers.** Artificial base pairs amenable to PCR amplification and aptamer development together with the natural ones: P, 2-amino-8-(1′-β-D-2-deoxyribofuranosyl)-imidazo[1,2-a]-1,3,5-triazin-4(8H)one; Z, 6-amino-5-nitro-3-(1′-β-D-2′-deoxyribofuranosyl)-2(1H)-pyridone; Ds, 7-(2-thienyl)-imidazo[4,5-b]pyridine; and Px, 2-nitro-4-propynylpyrrole. Hydrogen-bond stabilization for the Ds-Px pair has not been described.

In the course of sequence optimization after structure prediction, to increase the affinity for the targets, the program AptaMut (112) can provide clues for the potential effects of point mutations on affinity and structure. To enhance the resistance of RNA aptamers to chemical and enzymatic degradation, inclusion of modified nucleotides, such as 2′-*O*-methyl, 2′-fluoro, or other unnatural nucleotides (138, 139) in place of the natural 2′-OH ribose are common approaches. A step forward was introduced independently by Hirao and colleagues (140, 141), and Tan, Benner and coworkers (142), who incorporated modified nucleotides originally designed to form unnatural base pairs for synthetic xenobiology, into aptamers, increasing the resistance of the oligonucleotides to degradation (Fig. 8).

Among the challenges in translating aptamers against amyloidogenic proteins into therapeutic applications is also their potential toxicity, similar to other nucleic-acid-based drugs. For example, in the development of antisense therapy, acute hepatotoxicity of 2′-fluoro-modified antisense oligonucleotides was found in 6- to 8-week-old wild-type mice, in which the oligonucleotides were administered subcutaneously at 20 mg/kg three times a week (143). In contrast, in other studies, a 2′-fluoro nucleotide-stabilized siRNA was administered subcutaneously as a single, 30-mg/kg bolus for 2 years in a rat or at daily 7.5-mg/kg injections for 5 days in healthy volunteers without apparent toxicity (144). Similarly, antisense oligonucleotides containing 2′-O-methyl nucleotides were administered to cynomolgus macaques (*Macaca fascicularis*) at 20 mg/kg by subcutaneous injections for 9 months and to patients with hereditary transthyretin amyloidosis in a phase 2/3 clinical study at 300 mg for 15 months without cytotoxic effects (145). Another concern is that as is the case with antibodies (see, *e.g.*, an interesting report by Hatami *et al.* (146)), aptamers may cross-react with off-targets. In particular, aptamers against amyloid proteins might cross-react with functional human amyloids (147, 148) and disrupt their function, leading to cytotoxicity. Thus, in addition to characterization of their binding and efficacy, aptamers intended for *in vivo* use must be evaluated for their safety and be applied only if they have a sufficiently large therapeutic index. In this context, an advantage of aptamers as chemotypes for drug development is the ease with which they can be modified and subjected to structure–activity relationship studies compared with small molecules or protein biologics.

### Prediction of aptamer–protein interaction by machine learning and molecular docking

After determination of the secondary and tertiary structures of new aptamers, deciphering the aptamer–protein complex structure often is the next goal. Molecular docking of aptamers and target proteins on each other is a forecasting method that typically consists of searching all the potential binding modes between the pair of molecules *in silico* and scoring each complex based on its thermodynamic stability. As an example, a docking simulation of multiple aptamer–protein pairs was carried out to optimize the aptamer sequence using different proteins in combination either with RNA aptamers or with ribosomal RNA by Zhang *et al.* (149) using FTDock. The report was primarily methodological, but we expect that this methodology will be useful for future drug development.

Recently, a new Sequential Multidimensional Analysis algoRiThm for aptamer discovery (SMART-Aptamer) using HT-SELEX based on machine learning has been described (150). This system of molecular docking allowed obtaining high-affinity aptamers with low false-positive and false-negative rates. This new system coupled with modeling of the tertiary structure has high promise for optimization of the interaction mode between aptamers and amyloid–protein targets. However, although these approaches have helped optimizing aptamers in different cases, such as for human embryonic stem cells, epithelial cell adhesion molecule, and cell-surface vimentin (150), they have not yet been used in the amyloid field.

## Brain-targeting delivery of aptamers by exosomes and nanoliposomes

Oligonucleic acids are too large and negatively charged to pass through the blood–brain barrier (BBB). To exert the functions of amyloid-targeting aptamers in the CNS, the development of effective delivery systems is an important challenge. Since bare nucleic acid delivered systemically also would be subject to enzymatic degradation and may not even reach the BBB in sufficient amounts (151), innovative engineering of CNS-targeting delivery methods of aptamers using nanocarriers, such as exosomes or nanoliposome, has been a major focus in recent years.

Exosomes are nanovesicles, 30 to 200 nm in diameter, secreted by virtually all cell types and are thought to mediate intercellular and interorgan communication, as well as serve a mechanism for removal of cellular stressors (152, 153, 154). They can be used as a delivery mechanism when the cargo is expressed in cultured cells and the exosomes containing the cargo are isolated from the cell-culture medium.

**Figure 9:**
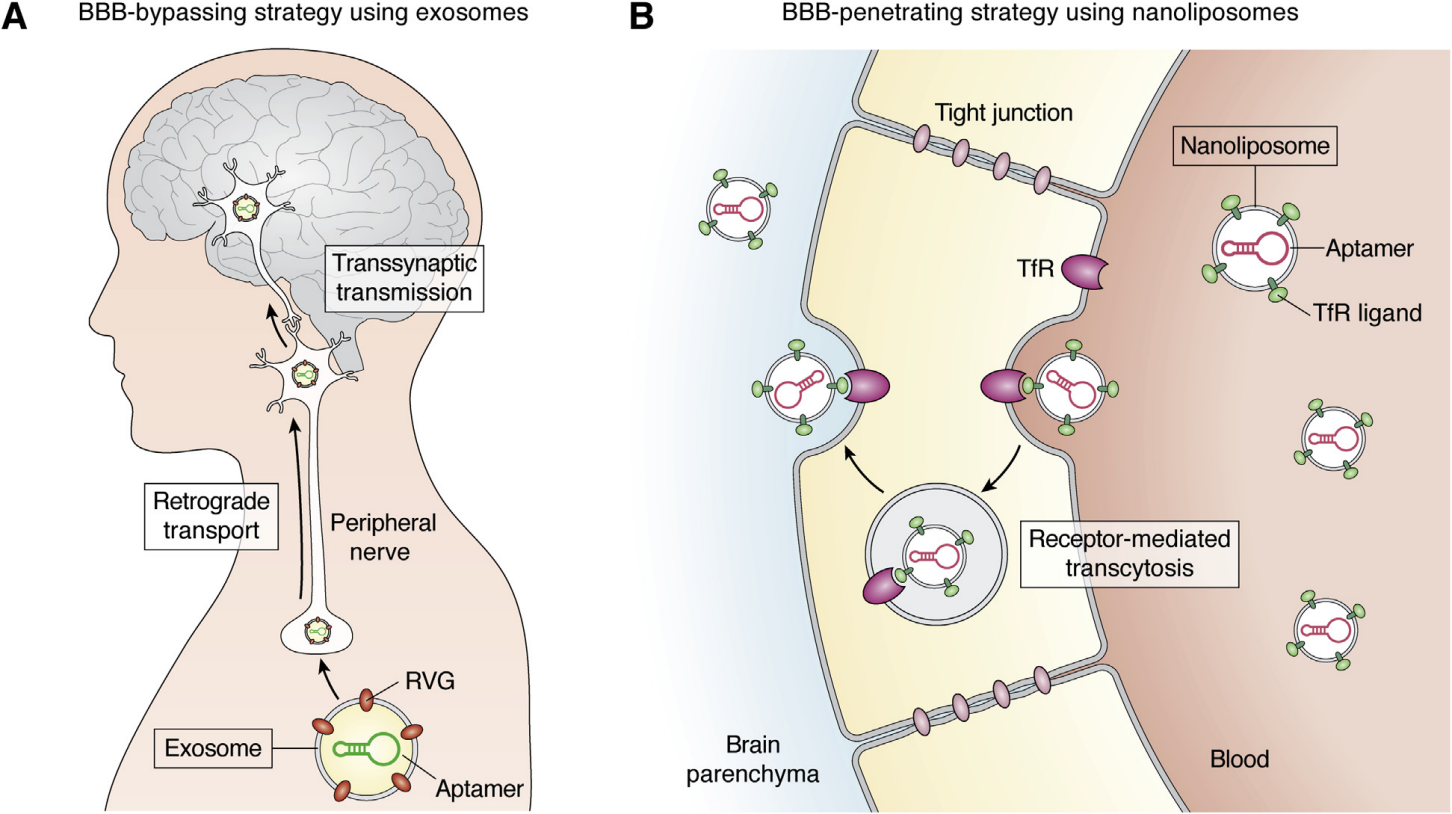
**Strategies for delivering aptamers into the CNS by encapsulation in nanovesicles.***A*, aptamers encapsulated in exosome expressing rabies virus glycoprotein (RVG) on their surface are retrogradely and transsynaptically transported from the peripheral nervous system into the brain, circumventing the BBB. *B*, aptamers included in nanoliposome carrying ligands of transferrin receptor (TfR) are transported from the blood to the brain parenchyma by receptor-mediated transcytosis.

A follow-up study on the DNA aptamer F5R1 (82) mentioned above revealed that it bound preferably to fibrillar, rather than monomeric αSyn (155). To investigate a therapeutic potential of F5R1, it was encapsuled, following polyethylenimine-assisted transfection, in exosomes isolated from the culture medium of HEK293 cells. It was then delivered into the brain of wild-type mice, which received intrastriatal injection of preformed recombinant αSyn fibrils prepared from recombinant αSyn, by taking advantage of a virus-transmission system (Fig. 9*A*) (155). The cells also expressed rabies virus glycoprotein (RVG) fused with lamp2B, which positions the RVG on the outer membrane of exosomes secreted by these cells. Intraperitoneal administration of the RVG-decorated exosomes containing F5R1 led to retrograde transport and transsynaptic transmission into the CNS through the axons and synapses of peripheral neurons, circumventing the BBB. The treatment decreased αSyn aggregation in the substantia nigra and ameliorated motor dysfunction in the treated mice (155). This pioneering work provided proof of concept for application of DNA–aptamer therapy delivered into the CNS using an innovative delivery system that bypasses the BBB, resulting in reduction of the aggregation, transmission, and toxicity of αSyn in a preclinical model of synucleinopathy.

A different aptamer-delivery method into the CNS using nanoliposomes was reported by McConnell *et al.* (Fig. 9*B*) (156). Like exosomes, nanoliposomes are nanovesicles, yet they are prepared synthetically, as opposed to the natural origin of exosomes, allowing a greater degree of control over their composition and cargo. Similar to exosomes, the nanoliposomes are enclosed by a lipid bilayer surrounding trapped water in which hydrophilic compounds can be transported. McConnell *et al.* encapsuled a dopamine-specific DNA aptamer, Da20m, into nanoliposomes conjugated on their surface by a different aptamer, specific for the transferrin receptor (TfR). TfR is expressed on endothelial cells in the luminal surface of the BBB. Binding of the anti-TfR aptamer to TfR followed by clathrin-mediated transcytosis of TfR allowed delivery of the nanoliposomes across the BBB and release of the antidopamine aptamer cargo on the CNS-parenchymal side. Compared with unencapsulated Da20m, which had little or no effect, systemic administration of Da20m *via* the functionalized nanoliposomes potently attenuated abnormal behavior of wild-type mice triggered by aberrantly increased extracellular dopamine following exposure to cocaine (156). This system appears to be highly promising, yet to our knowledge it has not been used yet for delivery of aptamers against amyloidogenic proteins.

To date, over 20 liposomal products have been approved by the FDA, including anticancer drugs and vaccine formulations against SARS-CoV-2 containing RNA molecules (157, 158). Encapsulation of RNA (and DNA) in liposomes promotes the biosafety and stability of the exogenous nucleic acids inside the body. Both exosomes and nanoliposomes are attractive delivery systems for aptamers. Exosome cargo and surface markers are easier to manipulate genetically, compared with those of nanoliposomes, yet nanoliposomes can be produced more easily on large scales (159). In addition, because exosomes are produced in cells and may contain unintended biological material, it is crucial to establish ways of controlling and monitoring their clinical safety if they are going to be approved for therapeutic applications (160). Although exosomes are less immunogenic than cell-based therapies, a concern in using them as drug carriers is that they may be recognized as nonself by the immune system and induce an immune response, potentially causing damaging inflammation. Should this become a problem, it could be overcome by extracting the patient’s own cells and preparing the exosomes using the patient-derived cells. Though more time-consuming and potentially expensive, such a procedure could be justified for life-saving treatments.

## Conclusions and future perspectives

In 2004, the first aptamer drug, the RNA aptamer Macugen (pegaptanib sodium), which acts as an antagonist of vascular endothelial growth factor, was approved by FDA for treatment of age-related macular degeneration. Several additional aptamers have been tested in clinical trials for cancer, cardiovascular disease, and eye diseases, yet to date, despite the growing number of reports on antiamyloid aptamers and the advances in their design and delivery discussed above, aptamers have not yet advanced to clinical trials in the neurodegenerative disease field.

To advance the development of aptamers targeting amyloidogenic protein for biomedical applications, several issues should be addressed. First, the conformational metastability and heterogeneity of the targets, particularly oligomers of amyloidogenic proteins, are major impediments for aptamer selection. This challenge can be addressed by covalent tethering of the protein monomers to create a more stable aptagen that mimics the metastable oligomers, as was demonstrated in the case of Aβ42 (58), by identification of conformations or sequences responsible for pathology, as was done for prion (98) or tau (70), and by competitive selection of target assemblies in combination with counterselection of other species, as was successfully carried out for αSyn (51).

Second, isolating aptamers with high affinity and specificity for the desired target is a labor-intensive and time-consuming process that is always accompanied by a level of uncertainty. Next-Generation sequencing and structural prediction programs coupled with AI and machine learning algorithms are promising approaches for increasing both the speed of the process and the likelihood of success. Application of these modern techniques has been limited in the amyloid field but is beginning to catch up. Additional advances are expected when detailed structures of aptamers and their complexes with their respective targets become available and support structure prediction algorithms. To our knowledge, to date, only two X-ray crystallography studies reporting such structures have been published (161, 162). However, the increasing popularity of high-resolution structural determination by cryo-electron microscopy suggests that additional data will be forthcoming. High-resolution structures and improved structural prediction technology not only will facilitate obtaining improved aptamers, but also will help clarify the molecular basis of target recognition by aptamers, including in the amyloid field.

Finally, the inability of aptamers to cross the BBB on their own can now be addressed by at least two delivery systems, including exosomes and nanoliposomes, as discussed above. Each approach has its advantages and limitations, yet we expect that future research will address the current shortcomings and lead to successful application of these techniques in delivering aptamers targeting amyloidogenic proteins into the CNS, as has been demonstrated recently for aptamer F5R1 in a mouse model of synucleinopathy (155). Recent advances in both computational and experimental approaches suggest that using aptamers as antiamyloid therapeutics is achievable in the near future.

## Conflicts of interest

The authors declare that they have no conflicts of interest with the contents of this article.
